# Supplementary material for: Real-World Data from a Refractory Triple-Negative Breast Cancer Cohort Selected Using a Clinical Data Warehouse Approach
Source: Cancers (Basel). 2021 Nov 21;13(22):5835. doi: 10.3390/cancers13225835 (PMC8616548; doi:10.3390/cancers13225835)
Supplement: Supplementary file 1 [file cancers-13-05835-s001.zip › cancers-1404109-supplementary.pdf]

cancers MDPI

Supplementary Materials: Real-World Data from a Refractory Triple-Negative Breast Cancer Cohort Selected Using a Clinical Data Warehouse Approach

Hana Kim, Hyo-Jung Kim, Hongsik Kim, Hye-Ryeon Kim, Hyunji Jo, Joohyun Hong, Ryul Kim, Ji-Yeon Kim, Jin-Seok Ahn, Young-Hyuck Im, Se-Kyung Lee, Haeyoung Kim, Soo-Yong Shin and Yeon-Hee Park

Table S1. Differences of clinical tumor, nodal stages between de novo, nonrefractory metastatic triple-negative breast cancer, and refractory metastatic triple-negative breast cancer after surgery, and unresectable triple-negative breast cancer due to progression during neoadjuvant chemotherapy.

Group	De novo mTNBC	Recurrence of mTNBC after Curative Surgery		
			Refractory mTNBC	
		Nonrefractory mTNBC	Refractory mTNBC after Surgery	Unresectable mTNBC d/t Progression during NAC
Patient, no.	**69**	131	207	44
cT stage				
T1	4	36	23	4
T2	13	76	119	17
T3	17	14	55	19
T4	24	1	9	4
Unknown	11	4	1	0
cN stage				
N0	5	55	56	4
N1	3	33	46	17
N2	20	25	62	19
N3	35	16	42	4
Unknown	6	2	1	0

Abbreviations: mTNBC, metastatic triple-negative breast cancer.

Table S2. Characteristic differences between patients with nonrefractory triple-negative breast cancer and refractory triple-negative breast cancer who could receive surgery with a curative aim.

No., (%)	Nonrefractory mTNBC	Refractory mTNBC
Patient, no.	131	207
Surgery type		
BCS	55	80
Mastectomy	76	127
Adjuvant RT		
Yes	89	156
No	42	51
Adjuvant chemotherapy		
Yes	101	102
No	30	105
Adjuvant chemotherapy regimen		
AC	10	9
AC-T	48	30
Capecitabine	1	17
CMF	13	9
FAC	21	19
T	3	8
Ohers	5	10

Abbreviations: mTNBC, metastatic triple-negative breast cancer; BCS, breast-conserving surgery; RT, radiation therapy; AC, Adriamycin/cyclophosphamide, AC-T, AC followed by paclitaxel or docetaxel; CMF, cyclophosphamide/methotrexate/fluorouracil; FAC, fluorouracil/Adriamycin/cyclophosphamide; T, paclitaxel or docetaxel.
